# Mesenchymal stem cell-derived exosomes protect beta cells against hypoxia-induced apoptosis via miR-21 by alleviating ER stress and inhibiting p38 MAPK phosphorylation

**DOI:** 10.1186/s13287-020-01610-0

**Published:** 2020-03-04

**Authors:** Jin Chen, Junqiu Chen, Yuanhang Cheng, Yunfeng Fu, Hongzhou Zhao, Minying Tang, Hu Zhao, Na Lin, Xiaohua Shi, Yan Lei, Shuiliang Wang, Lianghu Huang, Weizhen Wu, Jianming Tan

**Affiliations:** 1 Fujian Provincial Key Laboratory of Transplant Biology, 900th Hospital, Xiamen University, 156th XiErHuan Road, Fuzhou, 350025 China; 2 Organ Transplant Institute, 900th Hospital, Clinical Medical Institute of Fujian Medical University, 156th XiErHuan Road, Fuzhou, 350025 China; 3 Xiangyang Central Hospital, Hubei University of Arts and Science, Xiangyang, 441000 China

**Keywords:** Mesenchymal stem cells, Exosomes, Hypoxia, Beta cells, ER stress

## Abstract

**Background:**

Hypoxia is a major cause of beta cell death and dysfunction after transplantation. The aim of this study was to investigate the effect of exosomes derived from mesenchymal stem cells (MSCs) on beta cells under hypoxic conditions and the potential underlying mechanisms.

**Methods:**

Exosomes were isolated from the conditioned medium of human umbilical cord MSCs and identified by WB, NTA, and transmission electron microscopy. Beta cells (βTC-6) were cultured in serum-free medium in the presence or absence of exosomes under 2% oxygen conditions. Cell viability and apoptosis were analysed with a CCK-8 assay and a flow cytometry-based annexin V-FITC/PI apoptosis detection kit, respectively. Endoplasmic reticulum stress (ER stress) proteins and apoptosis-related proteins were detected by the WB method. MiRNAs contained in MSC exosomes were determined by Illumina HiSeq, and treatment with specific miRNA mimics or inhibitors of the most abundant miRNAs was used to reveal the underlying mechanism of exosomes.

**Results:**

Exosomes derived from MSC-conditioned culture medium were 40–100 nm in diameter and expressed the exosome markers CD9, CD63, CD81, HSP70, and Flotillin 1, as well as the MSC markers CD73, CD90, and CD105. Hypoxia significantly induced beta cell apoptosis, while MSC exosomes remarkably improved beta cell survival. The WB results showed that ER stress-related proteins, including GRP78, GRP94, p-eIF2α and CHOP, and the apoptosis-related proteins cleaved caspase 3 and PARP, were upregulated under hypoxic conditions but were inhibited by MSC exosomes. Moreover, the p38 MAPK signalling pathway was activated by hypoxia and was inhibited by MSC exosomes. The Illumina HiSeq results show that MSC exosomes were rich in miR-21, let-7 g, miR-1246, miR-381, and miR-100. After transfection with miRNA mimics, the viability of beta cells under hypoxia was increased significantly by miR-21 mimic, and the p38 MAPK and ER stress-related proteins in beta cells were downregulated. These changes were reversed after exosomes were pretreated with miR-21 inhibitor.

**Conclusions:**

Exosomes derived from MSCs could protect beta cells against apoptosis induced by hypoxia, largely by carrying miR-21, alleviating ER stress and inhibiting p38 MAPK signalling. This result indicated that MSC exosomes might improve encapsulated islet survival and benefit diabetes patients.

## Introduction

Diabetes affects more than 450 million people, which is a tremendous economic burden worldwide. Islet transplantation is the most effective treatment for brittle diabetes. It was reported that more than 1500 patients had undergone islet transplantation, and 50–70% of patients were free from insulin injections according to ISCT [1]. However, more than 50% of transplanted islets were destroyed or dysfunctional due to rejection, blood-mediated inflammatory reaction, and insufficient vascularization [2]. Some bio-artificial pancreas models, such as macro-encapsulated islets and bio-scaffolds, have been developed to separate islets from blood, but poor vascularity and hypoxia remain the main hurdles to overcome [3–6]. Hypoxia is a major cause of islet death and dysfunction after transplantation [7]. Islets are highly oxygen-consuming cells that are sensitive to oxygen conditions. They represent only 2% of the pancreas but consume more than 10% of the oxygen supply [8]. However, transplanted islets often suffer hypoxia due to poor vascularity and low oxygen tension in transplanted sites. It was reported that the pO_2_ in macro-encapsulated devices is only 2–3% [9], while it is nearly 5–6% in native pancreatic islets [10, 11]. The pO_2_ levels have an important role in beta cell differentiation and function [12]. A large amount of oxygen is required for beta cells to produce ATP for insulin secretion [13]. Hypoxia can decrease beta cell viability [8], deteriorate beta cell function [14], and downregulate PDX1 and MAFA, which cause dysfunction of beta cells [15, 16]. In fact, hyperglycaemia in diabetes patients increases the oxygen consumption of beta cells, resulting in hypoxia of beta cells, which causes beta cell apoptosis and dysfunction [17]. Thus, improving beta cell viability and function under hypoxic conditions is a pre-requisite for macro-encapsulated islets and benefits diabetes patients.

Mesenchymal stem cells (MSCs), which exhibit many bioactivities, were found to promote islet survival and function in vitro and in vivo, but the mechanism has not been fully clarified [18]. We and other researchers have demonstrated that MSCs can release a large amount of exosomes, especially in a hypoxic environment [19], which are important mediators of intercellular communication. Exosomes can carry membrane proteins, intracellular proteins, mRNAs, and miRNAs of MSCs, which can affect many pathophysiological functions of recipient cells, such as inhibiting immune cell proliferation and promoting angiogenesis. However, whether exosomes derived from MSCs can improve the survival of beta cells against hypoxia-induced apoptosis is still unknown.

In this study, the mouse beta cell line βTC-6 was used to demonstrate that hypoxia induced beta cell apoptosis, and it was found that MSC exosomes protected beta cells against apoptosis induced by hypoxia through microRNAs by alleviating ER stress and inhibiting p38 MAPK signalling.

## Materials and methods

### Cell culture

Human umbilical cord MSCs (UC-MSCs, passage 3) were supplied by the Fujian Provincial Stem Cell Application Engineering Technology Research Center. MSCs were cultured and characterized as described previously [20]. In brief, MSCs were cultured in Dulbecco’s modified Eagle’s medium (DMEM, HyClone, Logan, UT, USA) supplemented with 10% foetal bovine serum (FBS, Gibco, Grand Island, NY, USA) at 37 °C and 5% CO_2_, and CD34, CD45, CD73, CD90, and CD105 (BD Biosciences, San Diego, CA, USA) were analysed by FACS. The differentiation potential of osteocytes and adipocytes was determined. The mouse beta cell line βTC-6 was purchased from the Cell Bank of the Typical Culture Preservation Committee of Chinese Academy of Sciences (Shanghai, China) and maintained in high-glucose DMEM (HyClone, Logan, UT, USA) supplemented with 10% FBS at 37 °C and 5% CO_2_ with normal air. Hypoxic culture conditions were maintained at 37 °C, 2% O_2_, and 5% CO_2_ (Panasonic, MCO-18 M, Japan).

### Isolation and characterization of exosomes from conditioned medium of MSCs

MSCs were cultured in DMEM supplemented with 10% exosome-depleted FBS (VivaCell Biosciences, Shanghai, China) under normal culture conditions (37 °C, 5% CO_2_). Cells were washed twice with phosphate-buffered saline (PBS) when they reached 70–80% confluence and then cultured in serum-free medium under hypoxic conditions (2% O_2_, 5% CO_2_, 37 °C) for an additional 48 h. The conditioned medium (CM) was harvested and centrifuged at 2000×*g* for 10 min to remove dead cells and cell debris. After filtration with 0.22-μm filters (Millipore, Carrigwohill, County Cork, Ireland) to remove microvesicles (0.2–1 μm), the supernatant was concentrated by centrifugation at 4000×*g* for 1 h using a 30-kDa molecular weight ultracentrifugal filter device Amicon Ultra-15 (Millipore, Carrigwohill, County Cork, Ireland). Exosomes in concentrated CM were isolated by ultracentrifugation at 100,000×*g* for 1 h using a Beckman XPN-100 ultracentrifuge at 4 °C. The representative markers of exosomes CD9, CD63, CD81, HSP70, and Flotillin 1 (Abcam, Cambridge, MA, USA) were identified by WB. The structure of exosomes was analysed by transmission electron microscopy (TEM, Hitachi HT-7700, Japan). The particle size distribution and concentration of exosomes were measured with nanoparticle tracking analysis (NTA) at NanoFCM Bioscience (Xiamen, China) with a Flow NanoAnalyzer (Xiamen, China) as reported [21]. CD9, CD63, and CD81, as well as markers of MSCs, including CD73, CD90, and CD105, were further confirmed by FACS after incubation with 4 μm aldehyde sulphate beads (Life Technologies, Carlsbad, CA, USA).

### Cell apoptosis and viability assay

Cell viability was measured using the cell counting kit 8 (CCK-8, Monmouth Junction, NY, USA). Beta cells (βTC-6) were seeded in 96-well plates and cultured in medium with different concentrations of MSC-derived exosomes (0, 6.25, 12.5, 25, 50, 100, 200 μg/mL) under normoxic (37 °C, 5% CO_2_, 21% O_2_) or hypoxic (37 °C, 5% CO_2_, 2% O_2_) conditions for 48 h. Then, CCK-8 reagent was added, and the cells were incubated at 37 °C for 2–3 h. The OD value was detected at 450 nm using a Multiskan (Thermo Fisher, USA). Cell apoptosis was analysed by AO/PI staining or an annexin V-FITC/PI apoptosis detection kit (BD) with a FACSCalibur flow cytometer (BD). βTC-6 cells were seeded in 6-well plates and cultured under normoxic or hypoxic conditions in the presence or absence of MSC-derived exosomes (50 μg/mL) for 48 h. The cells were harvested and stained with AO/PI for 5 min or fixed with 1× binding buffer following incubation with 5 μL FITC-Annexin V for 15 min and 5 μL PI for 5 min. Apoptotic cells were detected by microscopy or FACS.

### Western blot analysis

Western blot (WB) analysis was performed as previously described [22, 23]. In brief, cells were washed twice with cold PBS and then lysed with fresh RIPA buffer containing a cocktail of protease inhibitors. A total of 30 μg of protein from each sample was separated on 10% sodium dodecyl sulphate (SDS) polyacrylamide gels and then transferred to a PVDF membrane. After incubation with primary antibodies against CD9, CD63, CD81, Flotillin 1, HSP70, CHOP, cleaved Caspase 3, cleaved PARP, Survivin, GRP78, GRP94, phospho-eIF2α, eIF2α, phospho-p38 MAPK, p38 MAPK (Abcam, Cambridge, MA, USA, 1:2000 dilution), or beta actin (Abcam, 1:10,000 dilution) in TBST with 5% BSA, the membranes were washed three times with 5% milk (10 min each time) and then incubated with horseradish peroxidase-conjugated goat anti-rabbit or mouse secondary antibodies. The signals were visualized with enhanced chemiluminescence reagents (ECL, Thermo Fisher Scientific, Rockford, IL, USA).

### MiRNA sequencing

MiRNAs contained in MSC-derived exosomes were determined by Illumina HiSeq (KangChen Bio-tech, Shanghai, China). In brief, total RNA from exosomes was prepared and quantified with a NanoDrop ND-100. Small RNA adapters were then ligated to the 5′ and 3′ ends of total RNA. After cDNA synthesis and amplification, the PCR-amplified fragments were purified from the PAGE gel, and the completed cDNA libraries were quantified by an Agilent 2100 Bioanalyzer. Cluster generation was performed on an Illumina cBot, and sequencing was performed on an Illumina HiSeq 2000 according to the manufacturer’s instructions.

### Analysis of the effects of miRNA mimics and inhibitors

According to the miRNA sequencing results of exosomes, the mimics and inhibitors of five of the most abundant miRNAs (miR-21, let-7 g, miR-100, miR-381, miR-1246) and the control were synthesized by Ribo Biotech (Guangzhou, China). Beta cells (2 × 10^4^ for each group) were transfected with miRNA mimics or control at 100 nM (based on the suggested doses in the instructions and references [24]) with Lipofectamine 2000 reagent (Invitrogen, Carlsbad, CA, USA) and then cultured under hypoxic conditions for 48 h. To further confirm the effects of miRNAs in exosomes, beta cells (2 × 10^4^ for each group) were transfected with miRNA inhibitors (100 nM) and then cultured in medium with MSC-derived exosomes under hypoxic conditions. The dose-dependent effect of a selected miRNA was determined. Beta cells were transfected with multiple doses of the selected miRNA (0, 10, 20, 30, 50, 100, 200 nM). Cell viability was detected by CCK-8 assay, and the relative proteins were detected by WB analysis.

### Statistical analysis

Statistics were analysed using GraphPad Prism 6 software. Quantitative data are presented as the means ± SD. Multiple-group comparisons were performed with one-way ANOVA and Tukey’s post hoc test. The differences between two groups were analysed with a *t* test. A *P* value less than 0.05 was considered statistically significant.

## Results

### Characterization of exosomes derived from umbilical cord MSCs

**Fig. 1:**
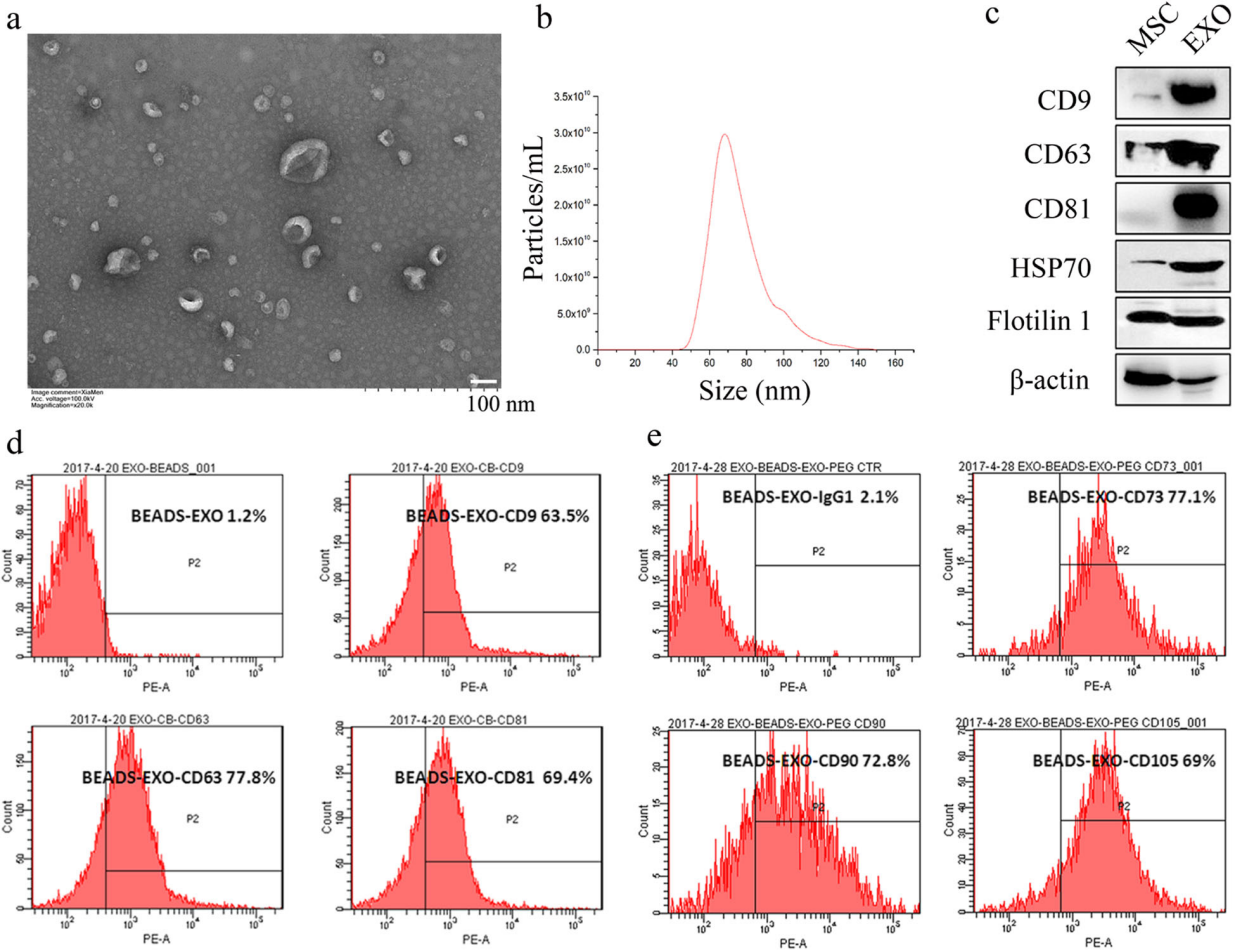
MSC exosome isolation and characterization. **a** Exosomes were analysed by transmission electron microscopy by negative staining of phosphotungstic acid. **b** The size distribution of exosomes was analysed by NTA with Flow NanoAnalyzer. **c** Exosome markers CD9, CD63, CD81, HSP70, and Flotillin 1 were identified by the WB method. **d** Exosome markers CD9, CD63, and CD81 and **e** MSC markers CD73, CD90, and CD105 were analysed by flow cytometry. EXO, exosomes

MSCs derived from the umbilical cord were first identified as we have previously described (Fig. S1) [20]. Next, exosomes derived from UC-MSC CM were enriched using a centrifugal ultrafiltration-based method and then isolated by ultracentrifugation. Exosomes were observed to be cup-shaped and 40–100 nm in diameter by transmission electron microscopy (Fig. 1a). The NTA results showed that the mean diameter of exosomes was 78.32 ± 14.84 nm with a concentration of 1.7 × 10^11^ particles/mL (Fig. 1b). WB analyses revealed that exosomes derived from UC-MSCs coexpressed CD9, CD63, CD81, HSP70, and Flotillin 1, all of which are reported as representative markers of exosomes (Fig. 1c). The positive expression of CD9 (63.5%), CD63 (77.8%), and CD81 (69.4%) in exosomes derived from UC-MSCs was further confirmed by flow cytometry assay (Fig. 1d). Moreover, the exosomes were also positive for MSC markers, including CD73 (77.1%), CD90 (72.8%), and CD105 (69%), indicating that the exosomes were indeed derived from MSCs as described (Fig. 1e).

### MSC-derived exosomes protect beta cells against hypoxia-induced apoptosis

**Fig. 2:**
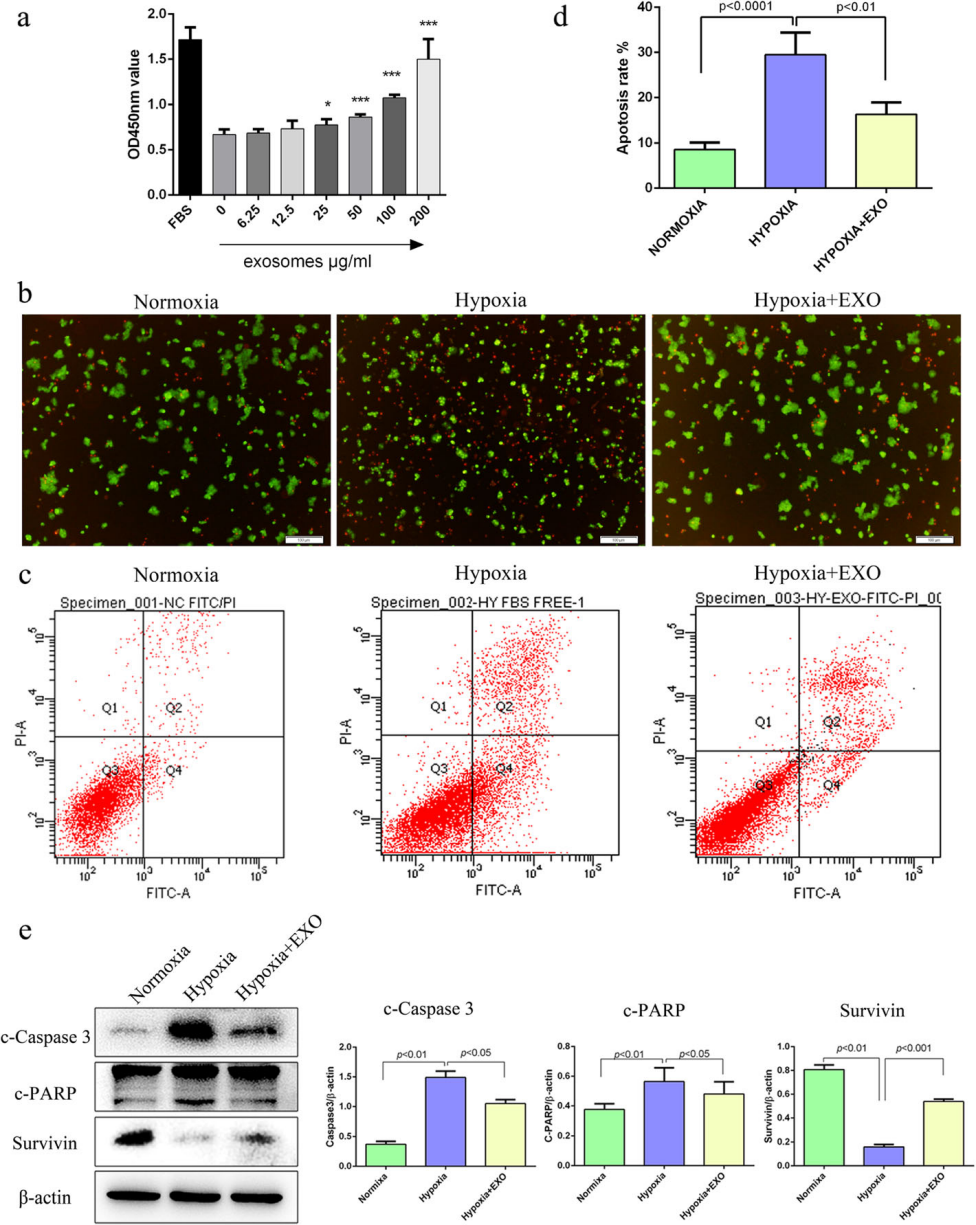
MSC exosomes attenuate beta cell apoptosis induced by hypoxia. **a** MSC exosomes promote beta cell survival in a dose-dependent manner. The beta cells were maintained under hypoxia (2% O_2_, 5% CO_2,_ 37 °C) and treated with different concentrations of MSC exosomes (0, 6.25, 12.5, 25, 50, 100, 200 μg/mL) for 48 h. Cell viability was detected by the CCK-8 method. **b**, **c** MSC exosomes attenuate hypoxia-induced beta cell apoptosis. Beta cells were cultured under normoxic (37 °C, 5% CO_2_, 21% O_2_) or hypoxic (37 °C, 5% CO_2_, 2% O_2_) conditions in the presence or absence of 50 μg/mL MSC exosomes for 48 h (hypoxia + EXO: hypoxia with 50 μg/mL exosomes). The viability of beta cells was determined by staining with AO/PI, the live cells are shown with green fluorescence, and the apoptotic cells are shown with red fluorescence (**b**). Cell apoptosis was analysed by an annexin V-FITC/PI apoptosis detection kit and flow cytometry (**c**, **d**). **e** The apoptosis-related proteins cleaved caspase 3 and PARP were downregulated, while the apoptosis inhibitor protein survivin was upregulated by MSC exosomes. The results are representative of three independent experiments. Data are expressed as the mean ± SD. *P < 0.05, ***P < 0.01 compared with the hypoxia control group (hypoxia without exosomes)

As has been reported, hypoxia can significantly induce apoptosis in beta cells. Thus, a cell apoptosis assay was first carried out with a FITC annexin V-PI apoptosis detection kit (BD) to confirm the effect of hypoxia on beta cells in our own study. Consistent with previous reports, our results also showed that the apoptosis of beta cells βTC-6 was remarkably increased under hypoxic conditions compared with normoxic conditions (Fig. S2). The apoptosis rate of beta cells βTC-6 under normoxic conditions was 10.9%, but it was as high as 28.5% under hypoxic conditions (Fig. S2b). The increased apoptosis of beta cells βTC-6 under hypoxic conditions was also evidenced by the enhanced cleavage of both Caspase-3 and PARP and downregulated expression of Survivin (Fig. S2c). To determine whether MSC-derived exosomes had any effect on hypoxia-induced apoptosis in beta cells, beta cells were cultured in the presence of different concentrations of exosomes (0, 6.25, 12.5, 25, 50, 100, 200 μg/mL) under hypoxia. As shown in Fig. 2a, while a low dose of MSC-derived exosomes (6.25, 12.5 μg/mL) did not affect the survival of beta cells under hypoxia, a high dose of exosomes (25, 50, 100, 200 μg/mL) significantly improved the survival of beta cells under hypoxia in a dose-dependent manner. Moreover, both AO/PI staining and flow cytometry showed that the presence of MSC-derived exosomes (50 μg/mL) significantly decreased the apoptosis of beta cells induced by hypoxia (Fig. 2b–d). The apoptotic rates of beta cells under normoxia and hypoxia with or without exosomes were 6.933 ± 0.318%, 12.33 ± 0.348%, and 20.27 ± 0.857%, respectively (Fig. 2c, d). Accordingly, the enhanced cleavage of both Caspase-3 and PARP by hypoxia was obviously alleviated, and the downregulated expression of Survivin was significantly recovered in the presence of exosomes (Fig. 2e). Collectively, these results indicate that MSC-derived exosomes exert a protective effect on hypoxia-induced apoptosis in beta cells.

### MSC-derived exosomes alleviate ER stress induced by hypoxia

**Fig. 3:**
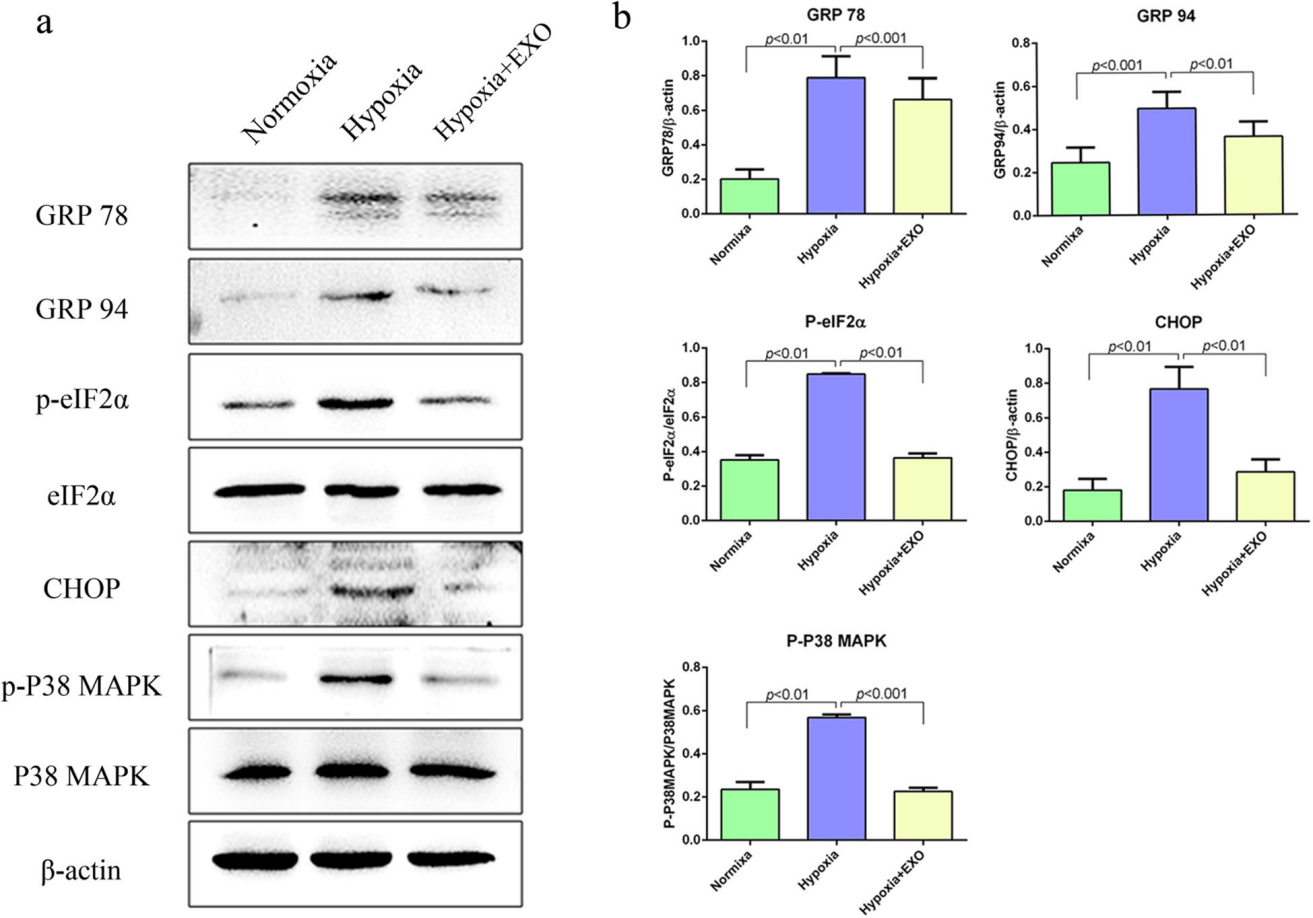
MSC exosomes alleviate hypoxia-induced beta cell apoptosis by attenuating ER stress and p38 MAPK activation. **a** ER stress-related proteins (GRP78, GRP94, P-eIF2α, and CHOP), P38 MAPK signalling, and apoptosis-related proteins (cleaved caspase 3, PARP, and survivin) were detected by WB. β-Actin was used as a loading control. ER stress-related proteins and the phosphorylation of P38 MAPK were inhibited by MSC exosomes. **b** Densitometry assay with ImageJ. NOR, normoxia group; HYP, hypoxia group; HYP + EXO, hypoxia with 50 μg/mL exosomes group

Hypoxia inhibits the formation of protein glycosylation and disulphide bonds, resulting in the accumulation of unfolded or misfolded proteins in the endoplasmic reticulum (ER). This condition is defined as ER stress, which reflects an imbalance between the cellular demand for ER function and ER protein folding ability. Prolonged or severe ER stress eventually results in cell apoptosis [25, 26]. To determine whether MSC-derived exosomes may protect beta cells against hypoxia-induced apoptosis by influencing ER stress, the expression of ER stress-related proteins, including eIF2α, CHOP, glucose-regulated protein 78 (GRP78), and GRP94, was detected by WB. We found that the expression of ER stress-related proteins was significantly upregulated under hypoxic conditions (Fig. S2 and Fig. 3a), suggesting that hypoxia could result in ER stress in beta cells. Interestingly, treatment with MSC-derived exosomes (50 μg/mL) significantly alleviated the upregulated expression of ER stress-related proteins induced by hypoxia in beta cells (Fig. 3a, b). Further mechanistic study showed that the activation of p38 MAPK signalling by hypoxia in beta cells was attenuated upon treatment with exosomes (Fig. 3a, b). Thus, our current data suggest that MSC-derived exosomes may alleviate ER stress resulting from hypoxia, which in turn protects beta cells against hypoxia-induced apoptosis.

### MiR-21 in MSC-derived exosomes improves beta cell survival

**Fig. 4:**
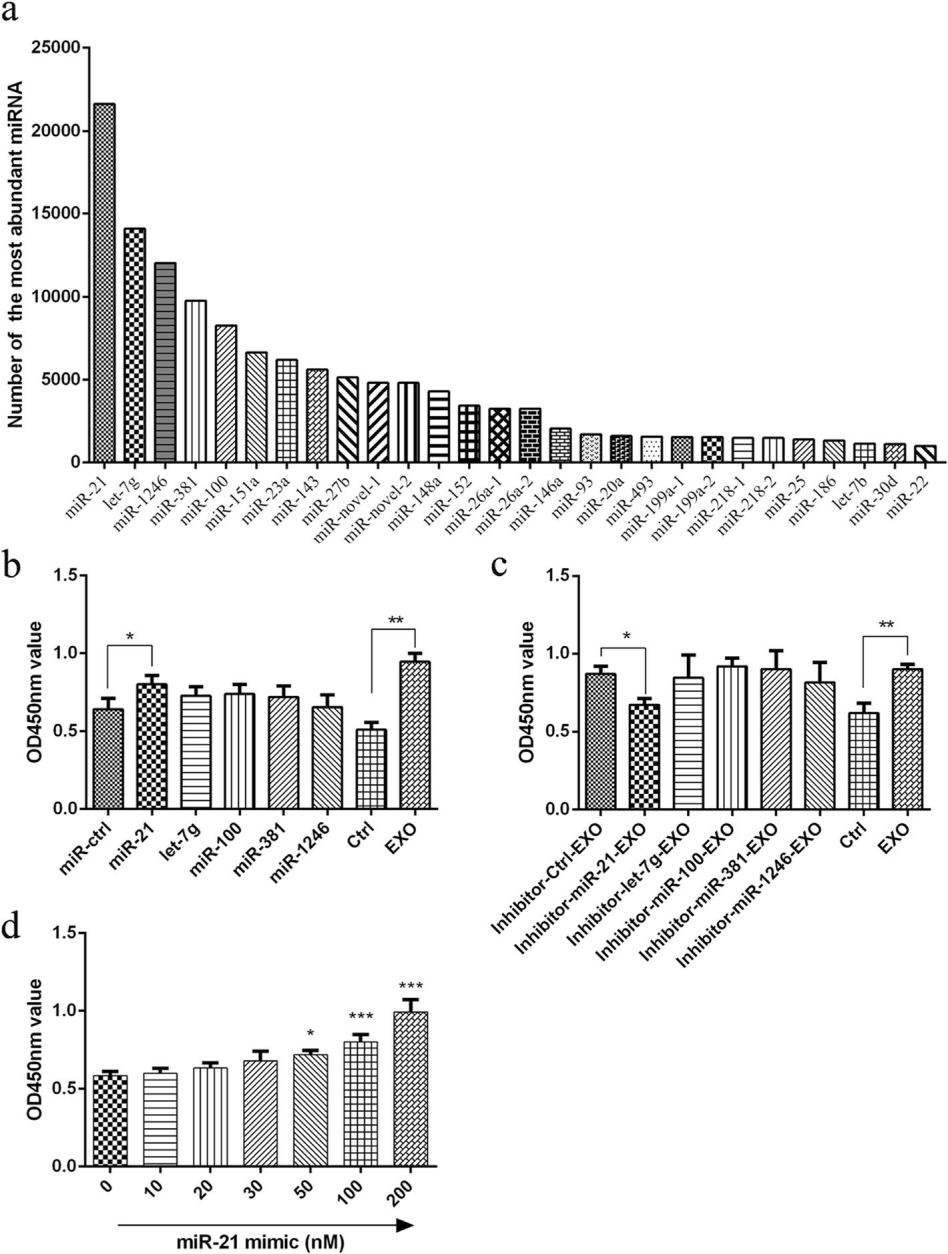
The effects of miRNAs in MSC exosomes on beta cells. **a** The miRNAs in MSC-derived exosomes were detected by Illumina HiSeq. MSC exosomes contained abundant miRNAs. MiR-21, let-7 g, miR-1246, miR-381, and miR-100 were the most abundant miRNAs in MSC exosomes. **b** The effect of the mimics (100 nM for each mimic) of the five most abundant miRNAs on beta cell survival in hypoxia. **c** The effect of the inhibitors (100 nM for each inhibitor) of the five most abundant miRNAs and their control miRNAs on beta cell survival in hypoxia. **d** Dose-dependent effects of miR-21 on beta cells in hypoxia. Cell viability was assayed by the CCK-8 method. OD values were detected at a wavelength of 450 nm. The results are representative of three independent experiments. Data are expressed as the mean ± SD. *P < 0.05, compared with the mimic control group

MiRNAs have emerged as important regulators of many biological processes. Since a large body of studies has revealed that exosomes contain abundant miRNAs, miRNA sequencing was applied in our study to analyse the miRNA expression profile in MSC-derived exosomes. The results demonstrated that thousands of different miRNAs were detected in MSC-derived exosomes. All miRNAs with amounts greater than 1000 are shown in Fig. 4a, in which miR-21, let-7 g, miR-1246, miR-381, and miR-100 were the most abundant miRNAs in MSC-derived exosomes. Next, the specific mimics or inhibitors of the most abundant miRNAs and their corresponding controls were used to analyse the effects of miRNAs in MSC-derived exosomes on beta cells. The results of the cell viability assay showed that among all mimics of the five most abundant miRNAs in MSC-derived exosomes, only miR-21 mimic significantly improved the survival of beta cells under hypoxia (Fig. 4b). Similarly, only pre-treatment with miR-21 inhibitor but not the other inhibitors, namely, let-7 g inhibitor, miR-1246 inhibitor, miR-381 inhibitor, and miR-100 inhibitor, significantly abrogated the protective effect of exosomes on the survival of beta cells in hypoxia (Fig. 4c). More importantly, the miR-21 mimic exhibited its protective effect on the survival of beta cells under hypoxia in a dose-dependent manner when used at 50 nM or more (Fig. 4d). Collectively, these data suggest that miR-21 may be the main mediator of the protective effect of MSC-derived exosomes on beta cell survival in hypoxia.

### MiR-21 alleviates ER stress and inhibits p38 MAPK signalling in beta cells

**Fig. 5:**
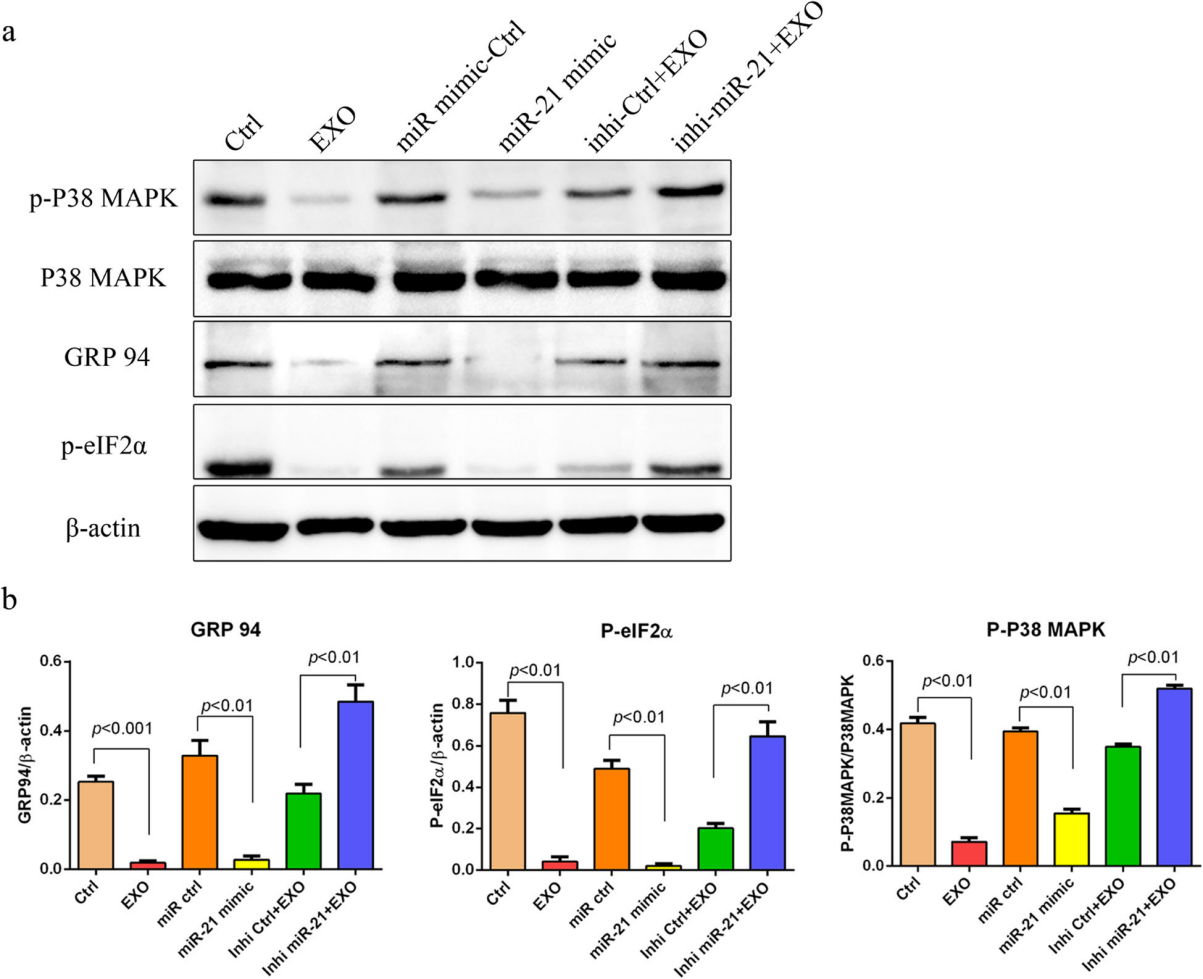
MSC exosomes alleviated ER stress and inhibited p38 MAPK phosphorylation via miR-21. **a** Beta cells were cultured under hypoxia (37 °C, 2% O_2_, 5% CO_2_) with or without MSC exosomes (50 μg/mL) for 48 h. Phosphorylation of p38 MAPK and the ER stress-related proteins GRP94 and P-eIF2α in beta cells, which were induced by hypoxia, were inhibited both by MSC exosomes and miR-21 mimics. After pre-treatment with the inhibitor of miR-21, the effects of MSC exosomes were reversed. β-Actin was used as a loading control. **b** Densitometry assay with ImageJ. Ctrl, beta cells were cultured under hypoxia in the absence of exosomes; EXO, beta cells were maintained under hypoxia in the presence of exosomes (50 μg/mL); miR-ctrl, beta cells were maintained under hypoxia in the presence of 100 nM miRNA mimic control; miR-21, beta cells were maintained under hypoxia in the presence of 100 nM miR-21 mimics; Inhi-ctrl, beta cells were maintained under hypoxia in the presence of exosomes (50 μg/mL) pretreated with miRNA inhibitor control (100 nM); inhi-miR-21, beta cells were maintained under hypoxia in the presence of exosomes (50 μg/mL) pretreated with miR-21 inhibitor (100 nM)

As has been demonstrated, p38 MAPK signalling was activated in beta cells under hypoxia (Fig. 3a, b). Given the reported role of miR-21 in p38 MAPK signalling [27], we then asked whether the protective effect of MSC-derived exosomes on beta cells under hypoxia may be attributed to the role of miR-21 via its effect on p38 MAPK signalling. As anticipated, while both exosomes and miR-21 mimic significantly inhibited the activation of p38 MAPK signalling by hypoxia, and pre-treatment with miR-21 inhibitor antagonized the effect of exosomes on p38 MAPK signalling (Fig. 5). Accordingly, the expression of ER stress-related proteins in beta cells induced by hypoxia was downregulated after treatment with MSC-derived exosomes or miR-21 mimics (Fig. 5). Again, the effect of MSC-derived exosomes on the expression of ER stress-related proteins in beta cells under hypoxia was completely abrogated by pre-treatment with miR-21 inhibitor (Fig. 5). Taken together, our data demonstrated that miR-21 in MSC-derived exosomes alleviated ER stress and inhibited p38 MAPK signalling in beta cells under hypoxia.

## Discussion

Exosomes are 40–100 nm diameter microvesicles released from various cells with the ability to transmit proteins, mRNA, and miRNA into receptor cells, playing a biological role [28]. Here, we isolated exosomes from UC-MSC-conditioned medium through ultrafilter centrifugation and ultracentrifugation [29]. The expression of CD9, CD63, CD81, Flotillin 1, and HSP70, which are characteristic markers of exosomes [29, 30], was characterized. It was also shown that the expression of CD73, CD90, and CD105, which are MSCs markers [31, 32], indicated that the harvested exosomes were derived from MSCs.

Oxygen is an important factor for beta cells. Hypoxia stress may play a role in the deterioration of beta cell function [17]. Low oxygen tension decreases beta cell viability and sensitivity to glucose [8]. Increasing the hypoxia tolerance of beta cells may help to improve beta cell viability and function and benefit diabetes patients. Herein, we demonstrated that the apoptosis of beta cells was increased in hypoxia. Hypoxia induces ER stress and p38 MAPK activation, which may increase beta cell apoptosis. MSC exosomes were used to protect beta cells against apoptosis induced by hypoxia. Our results showed that MSC exosomes significantly increased beta cell viability and inhibited beta cell apoptosis under hypoxia. The apoptosis-related proteins cleaved PARP and cleaved caspase 3 were downregulated, and the anti-apoptotic protein survivin was upregulated by MSC exosomes.

WB results also showed that MSC exosomes inhibited hypoxia-mediated ER stress and p38 MAPK signalling phosphorylation. Increasing ER stress in beta cells is one of the causes of islet physiological abnormalities, which trigger the autoimmune response in NOD mice [33, 34]. Moderate endoplasmic reticulum stress activates PERK- and p38-dependent apoptosis [25]. The anti-apoptotic proteins BCL-1 and Survivin decrease rapidly [25]. Apoptosis is induced through ROS-mediated ER stress via the JNK/p38 activation pathways in human cervical cancer cells [35]. Inhibition of PERK or p38 reduces cell death and apoptosis induced by a moderate dose of tunicamycin, an ER stress activator [25, 36]. Furthermore, p38 has been shown to regulate insulin secretion as well as the survival of pancreatic β-cells [37]. Inhibition of p38 phosphorylation enhanced beta cell insulin secretion and protected against oxidative stress-mediated beta cell failure [37].

To further explore how MSC exosomes affect beta cells, MSC exosomes were sequenced and found to be rich in multiple miRNAs, which may regulate the biological and pathological activities of recipient cells. We found that miR-21, let-7 g, miR-1246, miR-381, and miR-100 were the five most abundant miRNAs in MSC exosomes. The mimics and inhibitors of those miRNAs were used to clarify the effects of miRNAs in MSC exosomes. After treatment with the miR-21 mimic, the viability of beta cells under hypoxia was reduced significantly, while the other mimics showed no significant change. In addition, after treatment with an inhibitor of miR-21, the effect of MSC exosomes was reversed. This finding indicates that miR-21 in MSC exosomes (the most abundant miRNA in MSC exosomes) may have a major effect on beta cell survival.

MiR-21 is an important miRNA with various biological functions. MiR-21 was shown to attenuate oxygen and glucose deprivation-induced apoptosis in human neural stem cells by inhibiting p38 MAPK signalling [27] and could promote cell proliferation and inhibit apoptosis by the PTEN/PI3K/AKT signalling pathway [38, 39]. It was also reported that the deficiency of miR-21 causes cell apoptosis, necrosis, and vascular inflammation during atherogenesis [40]. Here, we confirmed that ER stress and p38 MAPK activation could be inhibited by miR-21 mimic, and the apoptosis of beta cells in hypoxia was reduced by miR-21 mimic.

## Conclusion

**Fig. 6:**
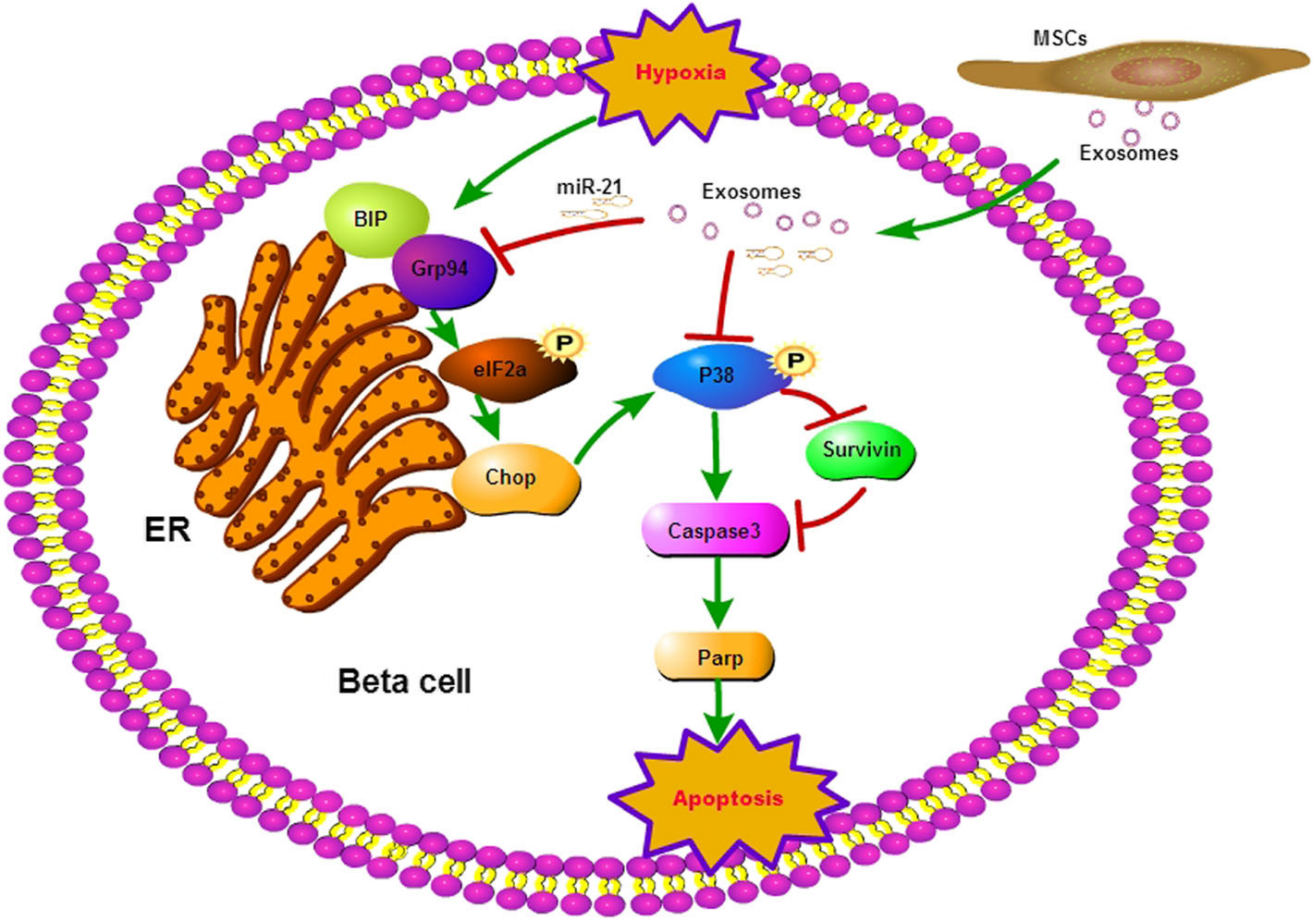
Diagram of the proposed model of MSC exosomes protecting beta cells against hypoxia-induced apoptosis. Hypoxia induces beta cell apoptosis by activating ER stress and p38 MAPK signalling and then upregulates the apoptosis-related proteins caspase 3 and parp, reducing survivin protein expression. MSCs could protect beta cells against apoptosis through exosomes carrying miR-21, which could alleviate hypoxia-mediated ER stress and inhibit hypoxia-induced p38 MAPK phosphorylation

In conclusion, the present study indicates that MSC-derived exosomes can protect beta cells against apoptosis via miR-21 by alleviating hypoxia-mediated ER stress and inhibiting p38 MAPK phosphorylation (Fig. 6). These results indicate that MSC exosomes might improve encapsulated islet survival and function and benefit diabetes patients.

## Supplementary information

**Additional file 1:Figure S1.** Characterization of human UC-MSCs. (a) UC-MSCs were positive for CD29, CD73, CD90, CD105 and negative for CD 14, CD34, CD45, HLA-DR. (b) UC-MSCs differentiation to osteocytes (alizarin red S staining) and adipocytes (Oil red O staining). **Figure S2.** Hypoxia induces beta cell apoptosis. Beta cells were cultured under normoxic (37 °C, 5% CO_2_, 21% O_2_) or hypoxic (37 °C, 5% CO_2_, 2% O_2_) conditions for 48 h. The viability of beta cells was determined by staining with AO/PI (magnification 100×) (a). Cell apoptosis was analysed by an annexin V-FITC/PI apoptosis detection kit and flow cytometry (b). Apoptosis-related proteins were assayed by the WB method (c). **Figure S3.** Hypoxia induces ER stress in beta cells. ER stress-related proteins were detected by WB method. Nor: beta cells culture in normoxia (37 °C, 5% CO_2_, 21% O_2_); HYP: beta cells cultured in hypoxia (37 °C, 5% CO_2_, 2% O_2_).

## Data Availability

The datasets during the current study are available from the corresponding author on a reasonable request.

## Abbreviations

MSCs: Mesenchymal stem cells
UC-MSCs: Human umbilical cord MSCs
EXO: Exosomes
ER stress: Endoplasmic reticulum stress
eIF2α: α-Subunit of eukaryotic translation initiation factor-2
p-eIF2α: Phosphorylation of eIF2α
P38 MAPK: p38 mitogen-activated protein kinase
GRP78/BIP: Glucose-regulated protein 78/binding-immunoglobulin protein
GRP94: Glucose-regulated protein 94
CHOP: C/EBP homologous protein (enhancer-binding protein homologous protein)
PARP: Poly ADP-ribose polymerase
C-CAS3: Cleaved caspase 3
ECL: Enhanced chemiluminescence
CCK-8: Cell counting kit-8
WB: Western blot
PDX1: Pancreatic and duodenal homeobox 1
MAFA: v-maf musculoaponeurotic fibrosarcoma oncogene homologue A
DMEM: Dulbecco’s modified Eagle’s medium
PBS: Phosphate-buffered saline
FACS: Fluorescence-activated cell sorter
ISCT: International Society Cell and Gene Therapy
NTA: Nanoparticle tracking analysis
