# Supplementary material for: Mesenchymal stem cell-derived exosomes protect beta cells against hypoxia-induced apoptosis via miR-21 by alleviating ER stress and inhibiting p38 MAPK phosphorylation
Source: Stem Cell Res Ther. 2020 Mar 4;11:97. doi: 10.1186/s13287-020-01610-0 (PMC7055095; doi:10.1186/s13287-020-01610-0)
Supplement: Supplementary file 1 — Additional file 1:Figure S1. Characterization of human UC-MSCs. (a) UC-MSCs were positive for CD29, CD73, CD90, CD105 and negative for CD 14, CD34, CD45, HLA-DR. (b) UC-MSCs differentiation to osteocytes (alizarin red S staining) and adipocytes (Oil red O staining). Figure S2. Hypoxia induces beta cell apoptosis. Beta cells were cultured under normoxic (37 °C, 5% CO2, 21% O2) or hypoxic (37 °C, 5% CO2, 2% O2) conditions for 48 h. The viability of beta cells was determined by staining with AO/PI (magnification 100×) (a). Cell apoptosis was analysed by an annexin V-FITC/PI apoptosis detection kit and flow cytometry (b). Apoptosis-related proteins were assayed by the WB method (c). Figure S3. Hypoxia induces ER stress in beta cells. ER stress-related proteins were detected by WB method. Nor: beta cells culture in normoxia (37 °C, 5% CO2, 21% O2); HYP: beta cells cultured in hypoxia (37 °C, 5% CO2, 2% O2). [file 13287_2020_1610_MOESM1_ESM.docx]

# Supplementary Information

**
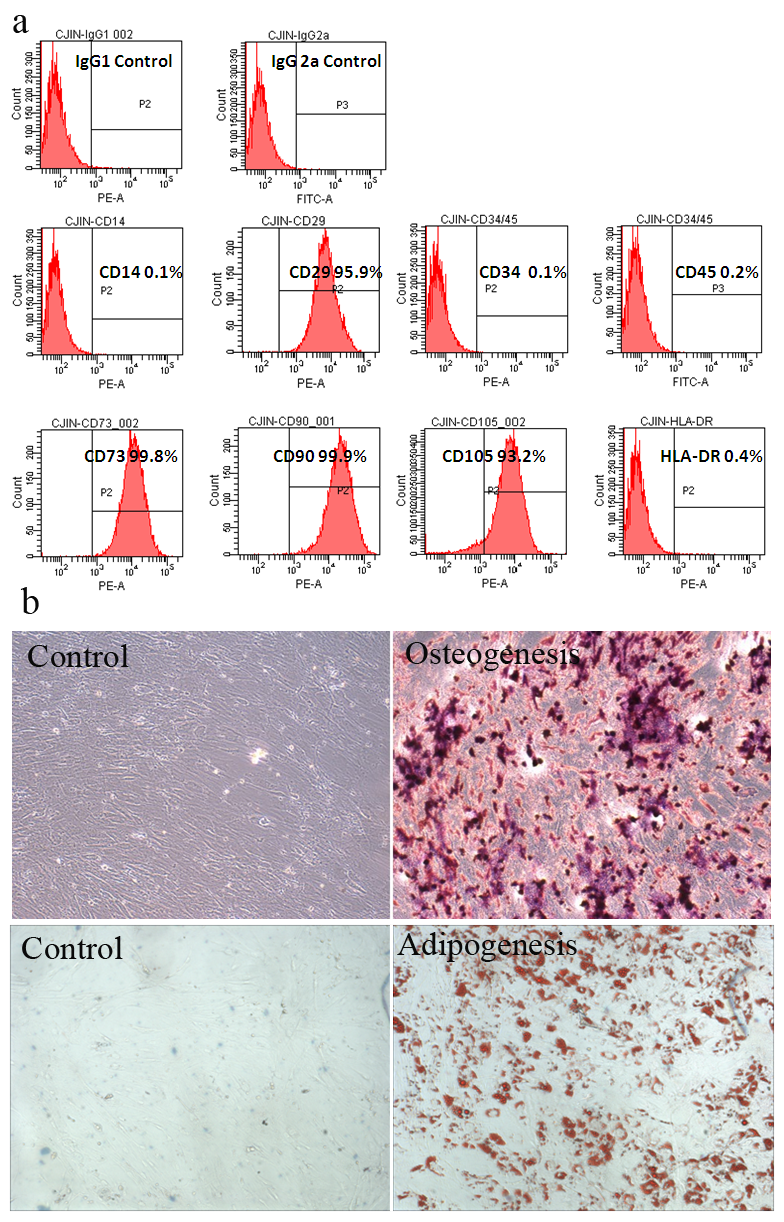
**

**Fig. S1. Characterization of human UC-MSCs.** (A) UC-MSCs were positive for CD29, CD73, CD90. CD105 and negative for CD 14, CD34, CD45, HLA-DR. (B) UC-MSCs differentiation to osteocytes (alizarin red S staining) and adipocytes (Oil red O staining).


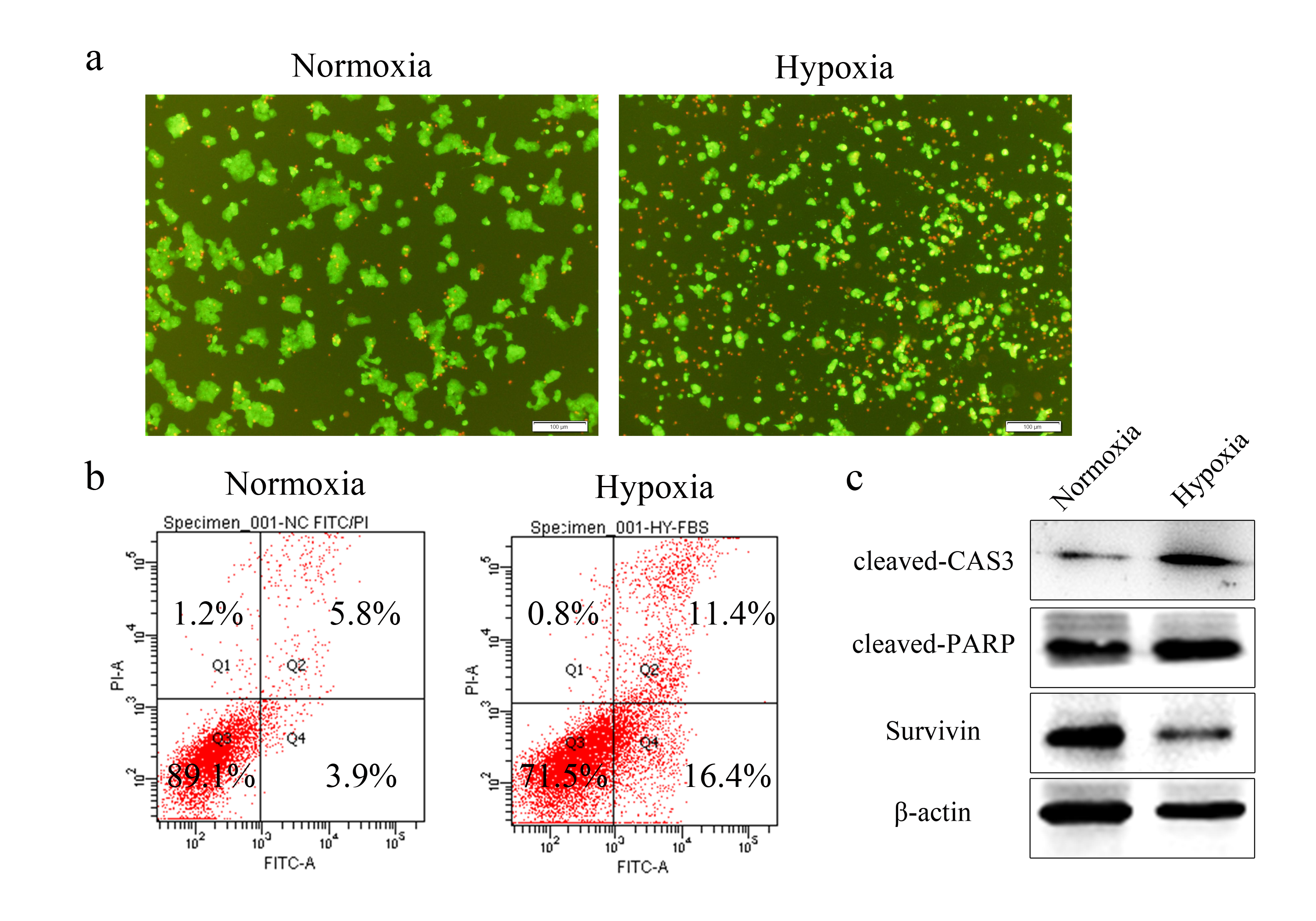


**Fig. S2. Hypoxia induces beta cell apoptosis.** Beta cells were cultured under normoxic (37 °C, 5% CO_2_, 21% O_2_) or hypoxic (37 °C, 5% CO_2_, 2% O_2_) conditions for 48 h. The viability of beta cells was determined by staining with AO/PI (magnification 100×) (A). Cell apoptosis was analysed by an annexin V-FITC/PI apoptosis detection kit and flow cytometry (B). Apoptosis-related proteins were assayed by the WB method (C).


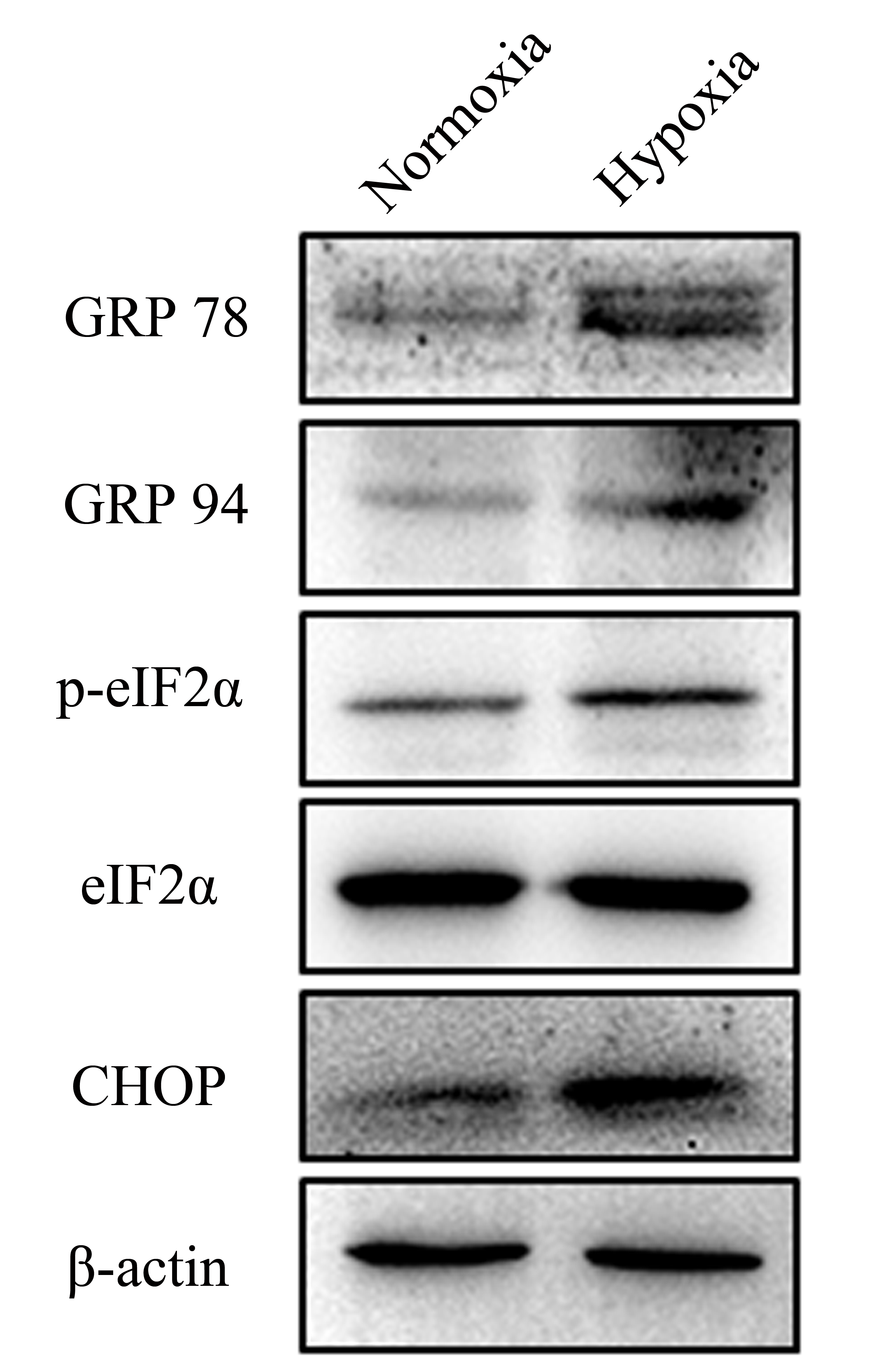


**Fig. S3. Hypoxia induces ER stress in beta cells.** ER stress-related proteins were detected by WB method. Nor: beta cells culture in normoxia (37 °C, 5% CO_2_, 21% O_2_); HYP: beta cells cultured in hypoxia (37 °C, 5% CO_2_, 2% O_2_).
